# Nonsense Mutation Inside Anthocyanidin Synthase Gene Controls Pigmentation in Yellow Raspberry (*Rubus idaeus* L.)

**DOI:** 10.3389/fpls.2016.01892

**Published:** 2016-12-19

**Authors:** Muhammad Z. Rafique, Elisabete Carvalho, Ralf Stracke, Luisa Palmieri, Lorena Herrera, Antje Feller, Mickael Malnoy, Stefan Martens

**Affiliations:** ^1^Research and Innovation Center, Fondazione Edmund MachSan Michele all’Adige, Italy; ^2^Genome Research, Department of Biology, Bielefeld UniversityBielefeld, Germany; ^3^Department of Developmental Genetics, Centre for Plant Molecular Biology, University of TübingenTübingen, Germany

**Keywords:** anthocyanidin synthase, yellow raspberry, pigmentation, mutation, complementation, pathway block

## Abstract

Yellow raspberry fruits have reduced anthocyanin contents and offer unique possibility to study the genetics of pigment biosynthesis in this important soft fruit. Anthocyanidin synthase (*Ans*) catalyzes the conversion of leucoanthocyanidin to anthocyanidin, a key committed step in biosynthesis of anthocyanins. Molecular analysis of the *Ans* gene enabled to identify an inactive *ans* allele in a yellow fruit raspberry (“Anne”). A 5 bp insertion in the coding region was identified and designated as ans^+5^. The insertion creates a premature stop codon resulting in a truncated protein of 264 amino acids, compared to 414 amino acids wild-type ANS protein. This mutation leads to loss of function of the encoded protein that might also result in transcriptional downregulation of *Ans* gene as a secondary effect, i.e., nonsense-mediated mRNA decay. Further, this mutation results in loss of visible and detectable anthocyanin pigments. Functional characterization of raspberry *Ans*/*ans* alleles via complementation experiments in the *Arabidopsis thaliana ldox* mutant supports the inactivity of encoded protein through ans^+5^ and explains the proposed block in the anthocyanin biosynthetic pathway in raspberry. Taken together, our data shows that the mutation inside *Ans* gene in raspberry is responsible for yellow fruit phenotypes.

## Introduction

Fruit pigmentation in raspberries (*Rubus idaeus* L., Rosaceae) is a complex phenomenon and one of the most important traits for breeding and consumer choice where a range of color patterns from deep purple to yellow exists. Anthocyanins and carotenoids are considered to be the main pigments involved in coloration of raspberries (de Ancos et al., 1999; Carvalho et al., 2013b). Anthocyanins are water soluble polyphenolic pigments responsible for the colors of many flowers (Chung et al., 2010; Luo et al., 2015; Sundaramoorthy et al., 2016), fruits (Saito et al., 1999; Debes et al., 2011; Liu et al., 2013; Ben-Simhon et al., 2015), and other plant tissues (Gould et al., 2000; Kim et al., 2004; Zhou et al., 2010). Their biochemical role in plants is not fully understood, however, they have been considered to protect tissues from biotic and abiotic stresses, delay in senescence, assist in photosynthetic machinery, delay over-ripening and increase shelf-life of fruits and act as scavengers of reactive oxygen intermediates (Zhang et al., 2013, 2016; Landi et al., 2015; Yousuf et al., 2016). They have also been described to have potential health beneficial effects in humans against cardiovascular and coronary heart diseases, beneficial effect on eye function properties, cancer, aging, inflammation, obesity, and neurodegenerative diseases (Cassidy et al., 2013; Lim et al., 2013; van der Heijden et al., 2016; Yousuf et al., 2016). Aside from the health benefits, anthocyanins play an important role as an indicator of fruit quality and consumer acceptance (Espín et al., 2007).

Genes encoding specific enzymes of the entire anthocyanin pathway have been well characterized in several plant species (Holton and Cornish, 1995; Broun, 2005; Lepiniec et al., 2006; Ferreyra et al., 2012). Structural and regulatory genes control the biosynthesis of anthocyanins while color mutants of structural and regulatory genes of the pathway have extensively been studied and characterized in many plant species (Kim et al., 2004; Kobayashi et al., 2004; Ma et al., 2009; Chung et al., 2010; Ben-Simhon et al., 2015).

Despite the interest in raspberry anthocyanins, little is known about the genetic control and their regulation processes during fruit development. It is supposed to be a complex trait involving not only the amount but also the type of pigments and co-pigments (Giusti et al., 1999; Castañeda-Ovando et al., 2009). Efforts have been made to identify and map the genes associated with anthocyanins in raspberry (Kassim et al., 2009; McCallum et al., 2010). Previous investigations carried out to explore the role of genes influencing pigmentation patterns propose that a dominant form of gene *T* plays crucial role in regulating the synthesis of anthocyanins (Crane and Lawrence, 1931). However, in yellow raspberries it seems to be a block of their biosynthesis (Määttä-Riihinen et al., 2004; Carvalho et al., 2013a). Many yellow varieties have originated from crosses between two red fruited varieties such as “Anne” (Amity × Glen Gerry), “Fall Gold” (NH-R7 × Taylor × *Rubus pungens* var. oldhamii), “Lumina” (Autumn Bliss × Tulameen), “Autumn Amber” (Polka × EMR earliest breeding line), “Zheltyi Gigant” (Maroseika × Ivanovskaya), and also some orange fruit varieties like “Orange Marie” [(Autumn Bliss × Fallgold) × Fallgold] and “Valentina” (EM6225/11 × EM5588/81). It has been discussed that there might be recessive form *tt* of gene *T* responsible for yellow phenotype of fruits (Crane and Lawrence, 1931), however, involvement of other genes has not been excluded which might affect the phenotype in the absence of dominant *T* allele (Britton et al., 1959; Macha, 1966; Jennings and Carmichael, 1975). Though the plants have been characterized in terms of chemical composition of fruits of yellow and red raspberry genotypes (Carvalho et al., 2013a,b) and the data obtained did not predict significant differences between red and yellow genotypes besides the presence or absence of anthocyanins.

Genetic studies have shown several genes such as chalcone synthase (*Chs*), flavanone 3*β*-hydroxylase (*F3h*), dihydroflavonol 4-reductase (*Dfr*), anthocyanidin synthase (*Ans*; synonym leucoanthocyanidin dioxygenase, *Ldox*), and UDPG-flavonoid-glycosyltransferase (*Ufgt*) control the formation of anthocyanins in fruits of Rosaceae species (Manning, 1998; Moyano et al., 1998; Takos et al., 2006; Ravaglia et al., 2013). Previous reports show that the amount of anthocyanins is strongly related to the level of expression of *Ans* gene (Almeida et al., 2007; Chen et al., 2012). In yellow raspberries there seems to be a block of anthocyanin biosynthesis, even though there is no evidence on where this block might occur (Määttä-Riihinen et al., 2004; Carvalho et al., 2013a) and knowledge of the molecular genetics is still scanty for this species. Against this backdrop, the present study provides new insights and improves the basic knowledge of origin of yellow raspberries as a result of genetic block in the anthocyanin pathway.

## Materials and Methods

### Plant Materials

Raspberry fruits of varieties “Anne” and “Tulameen” were collected in 2011 from 2 to 10 different plants per variety. Fruits were collected at different ripening stages from initial fruit formation (stage 1) to fully ripened fruits (stage 5) as described in Carvalho et al. (2013b). Young and old leaves of *Rubus* (“Anne”, “Tulameen”, “Golden Queen”, and “Heritage”) and *Fragaria* (*Fragaria* × *ananassa* and *Fragaria vesca*) were also collected. All samples were immediately frozen in liquid nitrogen and stored at -80°C until further use. Samples from different plants were kept and analyzed separately.

### DNA and RNA Extraction

Total RNA was isolated from leaves and different fruit stages using Spectrum Plant Total RNA kit (Sigma, Germany) according to the manufacturer’s instructions. Total RNA content and purity was assessed by Nanodrop 8000 (Thermo Scientific, USA) before proceeding to reverse transcription. Total RNA from 10 independent fruits of each stage was reverse-transcribed using SuperScript^®^ VILO^TM^ cDNA Synthesis Kit (Thermo Fisher Scientific, Waltham, MA, USA). Total genomic DNA was also extracted from young and old leaves according to instructions of NucleoSpin^®^ Plant II kit (Macherey-Nagel, Düren, Germany).

### Quantitative Real-Time PCR

Gene-specific primers for quantitative real-time PCR (qPCR) were designed using Primer Express 3.0 (PE Corporation, Foster City, CA, USA). Forward and reverse primer sequences for *Chs, F3h, Dfr, Ans, Ufgt*, and *Adh* (alcohol dehydrogenase) genes are listed in Supplementary Table S1. Real-time PCR was carried out in triplicates using C1000^TM^ Thermal Cycler CFX^TM^ (Bio-Rad Laboratories, Hercules, CA, USA) with the iQ^TM^ Sybr^®^ Green supermix (Bio-Rad Laboratories) under the following conditions: 98°C, 5 s followed by 44 cycles at 98°C 5 s, 58°C 5 s, 60°C 5 s, 76°C 10 s. After a denaturation step at 98°C for 30 s the melting curve analysis was done increasing the temperature of 0.2°C, from 65 to 95°C, each 10 s. The expression levels of different genes were normalized to the constitutive expression of *Adh* (GenBank Accession Number XM_004290519).

### Cloning of *Ans* Gene

Gene-specific primer “Ans-utr-fwd” and “Ans-utr-rev” (listed in Supplementary Table S1) were designed to amplify full length *Ans* gene. The amplified products from genomic DNA of both “Anne” and “Tulameen” were cloned into pCR^TM^4-TOPO^®^ vector by following the instructions of TOPO^®^ TA Cloning^®^ Kit for sequencing (Invitrogen). The cloned *Ans* gene from “Anne” and “Tulameen” was named as “pCR^TM^4-1840” and “pCR^TM^4-1835”, respectively. Then, *Ans* transcripts of “Anne” and “Tulameen” were cloned using the primer set (“Ans-orf-fwd” and “Ans-orf-rev”; Supplementary Table S1) specific to coding region. Coding regions of *Ans* of “Tulameen” and “Anne” were amplified and cloned into pENTR^TM^ D-TOPO^®^ vector by following the instructions of pENTR^TM^ Directional TOPO^®^ Cloning Kit (Invitrogen). The nucleotide sequences of cloned genes were verified by Sanger sequencing. Multiple sequence alignment was made using BioEdit (Hall, 1999).

### Copy Number of *Ans* Gene in *Rubus*

A qPCR, an alternative to Southern blot can successfully be utilized by normalizing any gene copies to that of the single copy gene by following the procedure as described earlier (Bustin, 2000; Solomon et al., 2008). For this purpose, full length “Anne” *Ans* gene cloned into pCR^TM^4-TOPO^®^ vector (“pCR^TM^4-1840”) was used for creating standard curve by following the instructions of Applied Biosystems (Life Technologies Corporation, Carlsbad, CA, USA). The standard curve was created by knowing the mass of “pCR^TM^4-1840” plasmid and running the respective serial dilutions (D1–D5) as described in protocol (http://www6.appliedbiosystems.com/support/tutorials/pdf/quant_pcr.pdf). For this purpose *Ans* target fragment was amplified by designing universal oligonucleotides (“RubUni-fwd” and “RubUni-rev”; Supplementary Table S1) aimed at species with known (*F. vesca*, n = x = 7; *Fragaria* × *ananassa* Duch, n = 4x = 28) and unknown (*Rubus*, n = x = 7) *Ans* copy number in comparison to the normalized plasmid reference. Reactions were performed using iQ^TM^ Sybr^®^ Green supermix (Bio-Rad Laboratories) by following the program, 98°C 10 s (denaturation), 49°C 5 s and 51°C 5 s (annealing) 76°C 10 s (extension), 39 cycles from denaturation to extension, 98°C 30 s (final extension) and melting curve 65–95°C with 0.2°C increment in C1000^TM^ Thermal Cycler CFX^TM^ Real-Time System (Bio-Rad Laboratories). All the reactions were carried out with the same set of established conditions in biological and technical triplicates.

### Transformation of *Arabidopsis*

The *Ans* gene from “Tulameen” (1242 bp) and “Anne” (1247 bp) present in pENTR^TM^ D-TOPO^®^ vector was sub-cloned into binary pLEELA vector by performing LR reaction (Invitrogen) and named as “pLEELA-1242” and “pLEELA-1247”, respectively. Both binary vectors containing *Ans* gene driven by *2x35S* promoter harboring *Bar* gene were introduced into *Arabidopsis ldox* mutant line (*tds4*-2; *tt18-1*) resistant for kanamycin marker using floral dip technique (Clough and Bent, 1998) via *Agrobacterium tumefaciens* strain GV3101. Seeds obtained were selected on half MS media (Murashige and Skoog, 1962) containing kanamycin (40 μg ml^-1^) and basta (25 μg ml^-1^) as selection markers. Homozygous F2 lines of F1 *Arabidopsis* seeds were obtained. The *Arabidopsis* transgenes were grown in growth chamber with the following conditions of 16/8 h light (100 μmol photons m^-2^ s^-1^), 70% humidity and 24°C temperature. Wild-type (Col-0) and control (*tds4-2*) plants were also grown with the same conditions in growth chamber. The transgenic status of several *Arabidopsis* lines obtained was evaluated by PCR amplification using “Ans-orf-fwd” and “Ans-orf-rev” primers (Supplementary Table S1) for *Ans* gene.

### Inducing Anthocyanins in *Arabidopsis* Seedlings

In order to evaluate the anthocyanins accumulation in complemented mutant lines of *Arabidopsis*, sterilized seeds were grown in half MS liquid media containing 5% sucrose under shaking (60 rpm). Sucrose stress promotes anthocyanins accumulation in emerging seedlings. Seeds were also placed in media without sucrose as control. Anthocyanin-based phenotype was observed in 5 days seedlings and 3 weeks old plantlets. Furthermore, plantlets were shifted to greenhouse for anthocyanin extraction and seed production. Anthocyanins were extracted in methanol (1% HCl) and subjected to HPLC analysis. The separation was accomplished under gradient conditions on a Nucleodur C18ec column (250/4; Macherey-Nagel) with solvent A 1% phosphoric acid in water and solvent B 1% phosphoric acid in acetonitrile. Gradient starts with 100% A to 50% A in 25 min, plateau of 3 min, up to 100% A in 7 min and final plateau of 5 min with flow rate of 1 ml min^-1^ and monitored at 280 and 515 nm.

### Establishment of Probe-Based Marker

Unlabeled oligonucleotides (“RubUni-fwd” and “RubUni-rev”) and a 15 bp TaqMan FAM dye-labeled probe (Supplementary Table S1) were designed using Primer Express 3.0 (PE Corporation) for detection of unique mutation inside *Ans* gene. All samples including non-template control (NTC) were run in triplicates in a volume of 12.5 μl containing 6.25 μl iQ Multiplex (Bio-Rad Laboratories), 0.25 μM each PCR primer, 0.5 μM FAM-probe and 20 ng genomic DNA or RNA/cDNA. Reactions were incubated in the C1000^TM^ Thermal Cycler CFX^TM^ Real-Time System (Bio-Rad Laboratories) for 3 min at 95°C (one cycle) followed by 39 cycles consisting of 10 s at 95°C, 10 s at 51°C and 30 s at 72°C.

## Results

### Expression Analysis of Anthocyanin Pathway Genes

**FIGURE 1:**
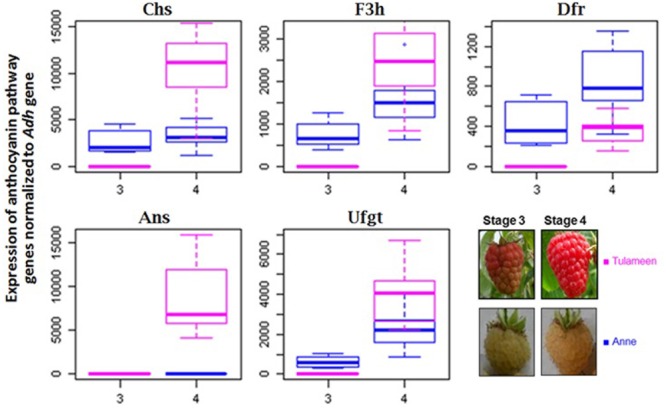
**Expression analysis of biosynthetic flavonoid pathway genes in cv. “Tulameen” and “Anne”.** Expression of *Chs.* chalcone synthase; *F3h.* flavanone 3β-hydroxylase; *Dfr*, dihydroflavonol 4-reductase; *Ans*, anthocyanidin synthase; *Ufgt* UDPG-flavonoid-glycosyltransferase at fruiting stage 3 and 4 in yellow variety “Anne” and red variety “Tulameen”, respectively. The inset picture shows an example of a red and a yellow raspberry at the respective fruit ripening stages. Box plots show the mean values of the expression level with standard deviation, while dots give the sample outline.

Structural genes of the anthocyanin biosynthetic pathway play key role in the biogenesis of anthocyanins in fruit tissues. Anthocyanins in raspberry fruits become visible starting from fruit stage 3 (“turning”). The expression pattern of the anthocyanin pathway genes of fruit development stages 3 and 4 in both varieties “Anne” and “Tulameen” are presented in **Figure 1**. *Chs, F3h, Dfr*, and *Ufgt* genes are upregulated from stage 3 to 4 at different levels in both varieties. In fruits of red variety “Tulameen” the apparent RNA accumulation of *Ans* gene is coordinated with the other biosynthetic genes examined (*Chs, F3h, Dfr, Ufgt*) as it shows a dramatic increase from stage 3 to 4. However, the increase in expression was not detected in yellow fruits of “Anne”. The expression profile shows that only the *Ans* gene is not upregulated onward fruit turning stage in “Anne” among all the other tested anthocyanin pathway genes (**Figure 1**). Therefore, further analysis of the *Ans* gene was performed to ascertain the possible block at the genetic level.

### *In silico* Mining and Genomic Structure of *Rubus Ans*

**FIGURE 2:**
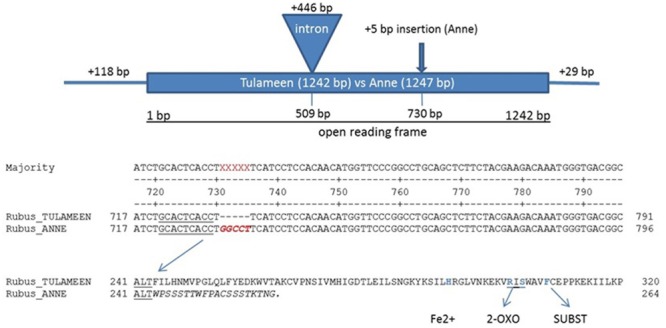
**Analyses of anthocyanidin synthase alleles in cv. “Tulameen” and “Anne”.** Comparison indicate a 5 bp insertion (ans^+5^) in the second exon at position 730 of “Anne” allele (ans) causing a frameshift and thus leading to a truncated protein in the yellow variety, missing conserved regions for substrate-, co-substrate, and iron binding sites. The *Ans* fragment from “Anne” and “Tulameen” includes the entire coding region with one 446 bp intron at position 509, and 118 bp of the 5′UTR and 29 bp of the 3′UTR.

*In silico* search of *Rubus* genome draft version 1.08 (cv. “Heritage”) and in house 454 EST library of fruit stages of “Tulameen” (unpublished data) with *Ans* gene of *Fragaria* × *ananassa* “Queen Elisa” (GenBank Accession Number AY695817) as template enabled the assembly of a putative *Ans* gene of *Rubus*. An *Ans* gene, spanning the full coding region was cloned with the help of assembled contigs (Supplementary Figure S1). Gene-specific primer were designed based on the sequence assembly for PCR amplification of a 1835 bp genomic *Ans* fragment from “Tulameen” that includes the entire coding region with one 446 bp intron, and 118 bp of the 5′UTR and 29 bp of the 3′UTR (GenBank Accession Number KX950789). Subsequently, the same approach was used to amplify the respective *Ans* gene of “Anne” (GenBank Accession Number KX950788) resulting in a 1840 bp amplicon. Direct comparison of these two alleles revealed a 5 bp insertion (GGCCT) in the second exon at position 730 bp of the “Anne” allele (ans; **Figure 2**). This insertion was designated as ans^+5^.

### Sequence Analysis of *Rubus Ans* Gene

A 1254 bp cDNA fragment of *Ri-Ans* was obtained by PCR amplification using primers designed from the predicted coding sequence using cDNA of “Tulameen” as template. The open reading frame (orf) contained 1242 bp, encoding a polypeptide of 414 amino acid (aa) residues and showing 92% similarity to the *Fragaria* × *ananassa* ANS protein. Comparison of *Ans* sequences revealed the same ans^+5^ in “Anne” cDNA as in genomic DNA sequences. This extra 5 bp sequence resulted in a frame-shift and creates a premature stop codon after 20 aa from this mutation point. It is predicted to result in a much shorter ANS protein of “Anne” of only 264 aa. The shorter “Anne” ANS protein lacks the essential conserved amino acids, known to be responsible for substrate and co-substrate binding (**Figure 2**), giving strong support for its inactivity and therefore the genetic block in the pathway.

Sequence alignment showed that the putative *Rubus* ANS grouped together with other known ANS from Rosaceae, but also other plant families and is separated from other flavonoid pathway 2-oxoglutarate-dependent dioxygenases (2-ODDs) such as flavonol synthase and flavanone 3*β*-hydroxylase (Supplementary Figure S2). It is obvious that most of the catalytical important residues are missing only in ANS of “Anne” in comparison to 2-ODDs (Supplementary Figure S2).

### Copy Number Analysis of *Ans* Gene

As the genome of *Rubus* is not fully sequenced yet (89%; Ward et al., personal communication) estimation of *Ans* gene copies is desirable. Here, in order to determine the copy number of *Ans* gene in *Rubus* we exploited the genome of diploid species *F. vesca* (2n = 2x = 14) with a single copy of *Ans* gene, and used *Fragaria* × *ananassa* Duch (octoploid; 2n = 8x = 56) with four copies of *Ans* gene (Almeida et al., 2007) as an additional endorsement to the methodology. To determine the genomic complexity, mass of “pCR^TM^4-1840” plasmid (5795 bp) was determined as 6.35e-18 g. Then, high quality standard curve was obtained by making serial dilutions (D1–D5). Similarly, mass of haploid genomes of *F. vesca* (n = x = 7; 120 Mb) and *Fragaria* × *ananassa* Duch (n = 4x = 28; 349 Mb) and *Rubus* (n = x = 7; approximately 150 Mb) was calculated as 3,288e-13 g, 2,630e-13 g, and 7,650e-13 g, respectively. Now, *Ans* target from *Rubus* and other reference species was put under comparative copy number analysis to the normalized single *Ans* copy (“pCR^TM^4-1840”). The corresponding ratio of *Ans* gene copies in haploid genome of *F. vesca, Fragaria* × *ananassa*, and different raspberry varieties is given in **Table 1**. It is evident that average numbers of *Ans* gene copies in *Rubus* varieties are closely correlated to the known *Ans* copies in *F. vesca* genome. Haploid genome of all the tested *Rubus* varieties (“Anne”, “Golden Queen”, and “Heritage”) contain single copy of *Ans* gene in comparison to haploid genome of *F. vesca* normalized to that of the single *Ans* copy gene. Furthermore, *F. vesca* and all the raspberry varieties present the same ratio (single copy of *Ans* gene) in comparison to the additional reference *Fragaria* × *ananassa* with four *Ans* copies (**Table 1**). Based on the results, single copy number of *Ans* gene in *Rubus* is suggested.

**Table 1:** Copy number of *Ans* gene in *Rubus* genome.

Rosaceae members	Mass of haploid genome (g)	Average *Ans* gene copies ± SD	Ratio w.r.t *Fragaria vesca* reference	Ratio w.r.t. *Fragaria* × *ananassa* reference
*Fragaria* × *ananassa*	2,6e-13	302714 ± 52332	–	4
*Fragaria vesca*	3,3e-13	76850 ± 5971	1	–
*Rubus idaeus* cv. Anne	7,6e-13	87050 ± 16858	1	1
*Rubus idaeus* cv. Golden Queen	7,6e-13	81971 ± 655	1	1
*Rubus idaeus* cv. Heritage	7,6e-13	64138 ± 3583	1	1

*Corresponding ratio scored to give integer copy number of *Rubus Ans* gene in Anne, Golden Queen, and Heritage with respect to (w.r.t.) *Fragaria vesca* and *Fragaria* × *ananassa* references in haploid genome of the Rosaceae members. Average numbers of *Ans* gene copies are presented along-with standard deviation values.*

### Complementation of *Arabidopsis ldox* Mutant

**FIGURE 3:**
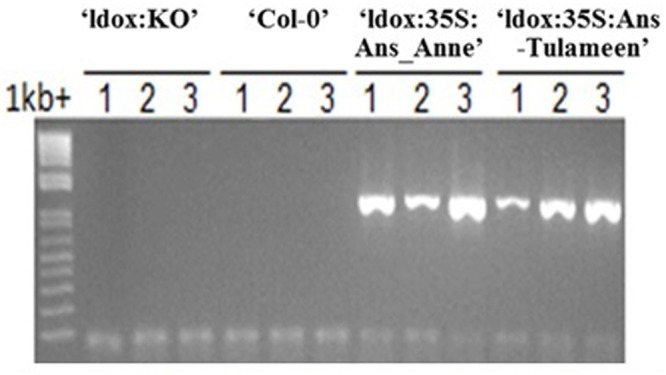
**PCR analysis, detecting *Ri-Ans* transgenes from “Anne” and “Tulameen”.** Independent transgenic (‘ldox::35S:Ans_Anne’ and ‘ldox::35S:Ans_Tulameen’) and independent non-transgenic (‘Col-0’ and ‘ldox:KO′) *Arabidopsis* lines.

**FIGURE 4:**
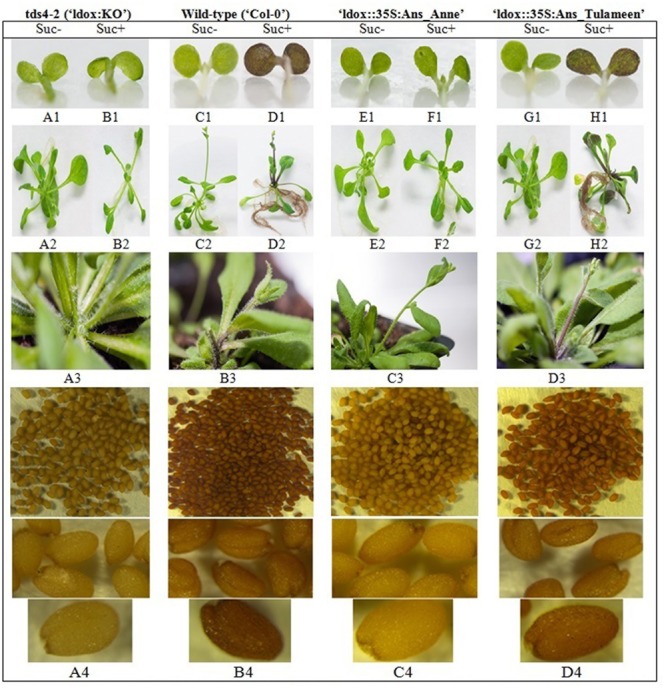
**Flavonoid phenotype of *Arabidopsis* transgenes in comparison to wild-type (′Col-0′) and *Idox* knockout line *(tds4-2; ‘*ldox:KO’).** Control, wild-type, ‘ldox::35S:Ans_Anne’ and ‘ldox::35S:Ans_Tulameen’ seedlings grown in half MS without sucrose (Suc-), 5 days old seedlings (**A1, C1, E1, G1**) and 2 weeks old plantlets (**A2, C2, E2, G2**), respectively. Control, wild-type, ‘ldox::35S:Ans_Anne’ and ‘ldox::35S:Ans_Tulameen’ seedlings grown on half MS with 5% sucrose (Suc+), 5 days old seedlings (**B1, D1, F1, H1**) and 2 weeks old plantlets (**B2, D2, F2, H2**), Respectively. Formation of anthocyanins was also obtained in 4 week old plants wild-type **(B3)** and ‘ldox::35S:Ans_Tulameen’ **(D3)** in comparison to control ‘ldox:KO’ **(A3)** and ‘ldox::35S:Ans_Anne’ **(C3)** lines. The seed coat color associated with presence of PA **(B4,D4)** or in absence of PA **(A4,C4)**.

Transgenic lines obtained from *ldox* mutant of *Arabidopsis* harboring *Ri-Ans* coding sequences from “Anne” and “Tulameen” driven by the constitutive *CaMV35S* promoter were termed as “ldox::35S:Ans_Anne” and “ldox::35S:Ans_Tulameen”, respectively, in connection to control *tds4-2* (“ldox:KO”) line. The transgenic status of *Arabidopsis* lines was confirmed by PCR amplification of *Ri-Ans* genes using DNA as template (**Figure 3**). Eight of 13 “ldox::35S:Ans_Tulameen” lines and five out of six “ldox::35S:Ans_Anne” lines were tested for functional activity. One representative line is shown from all genotypes (**Figure 4**).

Wild-type “Col-0” shows anthocyanins accumulation in 5 days old seedlings in response to sucrose stress induction as compared to seedlings with no anthocyanins in sucrose-less media. The control “ldox:KO” line could not produce anthocyanins in seedlings, irrespective of sucrose treatment. However, sucrose-mediated, wild-type-like anthocyanin phenotype of “ldox::35S:Ans_Tulameen” lines can clearly be noticed in hypocotyl and cotyledons of developing 5 days old seedlings. On the other hand, “ldox::35S:Ans_Anne” did not complement the *tds4-2* phenotype (**Figures 4A1–H1**). The absence of anthocyanins in “ldox::35S:Ans_Anne” line clearly demonstrates the non-functional status of “Anne” ANS protein in contrary to functional “Tulameen” ANS. None of the *Arabidopsis* lines presents phenotype with anthocyanins accumulation in their 5 days old seedlings in sucrose-less media. The phenotype of 2 weeks old plantlets in growth chamber in response to sucrose induction (**Figures 4A2–H2**) and 4 weeks old plants remained unchanged, i.e., “Col-0” and “ldox::35S:Ans_Tulameen” lines depict the presence of anthocyanins while “ldox:KO” and “ldox::35S:Ans_Anne” lines remained anthocyanin-less (**Figures 4A3–D3**).

Homozygous lines of “ldox::35S:Ans_Tulameen” showed a restored characteristic brown seed color as found in the wild-type “Col-0” whereas “ldox::35S:Ans_Anne” have the same yellowish color as the “ldox:KO” control line. The brown seed coat color of “ldox::35S:Ans_Tulameen” lines is associated with the presence of proanthocyanidins as found in the wild-type while seed coat of “ldox::35S:Ans_Anne” lines is yellow which is more likely comprised of mainly flavonols as in control “ldox:KO” line (**Figures 4A4–D4**). Furthermore, HPLC analysis showed the presence and a similar pattern of anthocyanins from extracts of all “ldox::35S:Ans_Tulameen” lines with wild-type *Arabidopsis* on the contrary to the plants of “ldox::35S:Ans_Anne” and “ldox:KO” lines (Supplementary Figure S3). Hence, functional characterization and complementation *in planta* provide an additional strong proof of inactive ANS protein in “Anne” as compared to functional ANS protein in “Tulameen”.

### Development of DNA/RNA-Based Probe Marker

**FIGURE 5:**
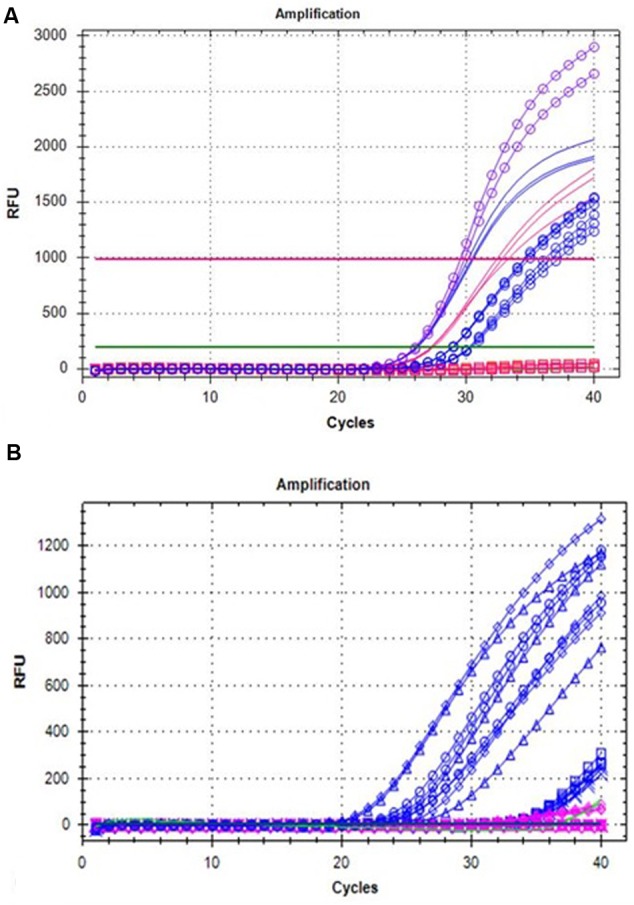
**(A)** Probe analysis on genomic DNA samples of “Anne” (blue) and “Tulameen” (pink) for the 5 bp insertion (ans^+5^). Probe assay on both genomic DNA (blue circles), ‘pCR^TM^4-1840’ (violet circles) of “Anne” and genomic DNA, ‘pCR^TM^4-1835’ (pink squares) of “Tulameen”. Genomic DNA samples amplified from “Anne” (blue lines) and “Tulameen” (pink lines) without probe in reaction mix. **(B)** Probe analysis on different fruit stages of “Anne” (blue) and “Tulameen” (pink) for the 5 bp insertion (ans^+5^). Transcripts from different fruit stages of “Anne” exhibits fluorescent signals (blue) but none of the “Tulameen” fruit stage (pink) as indicated by the diamond (Stage 1), cross (Stage 2), triangle (Stage 3), sequence (Stage 4), and circle (Stage 5). NTC is indicated along the baseline (green).

A big germplasm collection of *Rubus* is available at various platforms worldwide. It would be of a great interest in breeding programs to screen the *Rubus* varieties for Anne-like mutation inside *Ans* gene. Therefore, a DNA- or RNA-based probe marker was developed to detect ans^+5^ mutation. The probe was not only tested on independent samples of genomic DNA but also on RNA/cDNA of different ripening stages of fruits of “Anne” and “Tulameen”, respectively. In order to confirm the probe specificity, full length *Ans* gene from genomic DNA of the both varieties cloned into pCR^TM^4-TOPO^®^ vector (“pCR^TM^4-1835” and “pCR^TM^4-1840”) was also included in the analysis. As probe is designed on the basis of mutation inside *Ans* gene, it will not work on the samples that do not contain the mutation. Therefore, to validate the amplification, same primers were also tested on genomic DNA template of “Anne” and “Tulameen” in reaction mix exempting probe. The probe assay, as indicated in **Figure 5A**, shows that FAM dye-labeled probe containing 3 bp of ans^+5^ (GGCCT) was successfully applied to “Anne” *Ans* (“pCR^TM^4-1840”; violet circles) but not on “Tulameen” *Ans* (“pCR^TM^4-1835”; pink squares on baseline). Similarly, the FAM probe applied on various independent samples based on genomic DNA gave fluorescent signals for all the “Anne” samples (blue circles) but none of the “Tulameen” sample (pink square on baseline). However, not only “Anne” (blue lines) but all the “Tulameen” samples (pink lines) based on genomic DNA showed the amplification without probe (**Figure 5A**). The fluorescently labeled probe applied on RNA/cDNA also detected the mutation from the fruit stages of “Anne” (blue lines as shown in **Figure 5B**) and none of the fruit stage of “Tulameen” (pink) showed probe-based signals. NTC is also indicated along the baseline (green) as “Tulameen” samples. So it is clear from the probe analysis that the FAM probe is specific for mutation/insertion in “Anne” but work neither on genomic DNA nor RNA/cDNA of “Tulameen” samples (**Figures 5A,B**).

## Discussion

The current study provides new details in view of exploring the genetic mechanism controlling the biosynthesis of flavonoids, in particular anthocyanins, in raspberries. Raspberries with yellow or orange pigmentation, which have been described already more than a century ago (Card, 1898), are most probably based on anthocyanin pathway mutants. So far the knowledge is that homozygous recessive alleles *tt* of gene *T* are considered to play a fundamental role in determining the yellow color (Crane and Lawrence, 1931; Jennings and Carmichael, 1975). Whether this locus is related to *Ans* or not cannot be stated here. In the present work, the most sold red fruiting raspberry “Tulameen” was included for comparative study with the important pale yellow fruiting variety “Anne” to elucidate the phenomenon involved in pigmentation. Red raspberry “Tulameen” was selected from a cross “Nootka” × “Glen Prosen”, both bearing red fruits (Daubeny and Anderson, 1991). Yellow variety “Anne” has been selected from a controlled cross of two red varieties, “Amity” × “Glen Gerry” (Swartz et al., 1998); however, the authors did not provide any details on the inheritance of yellow pigmented fruits. In a recent study, biochemical analysis of various red and yellow raspberries including “Tulameen” and “Anne” gave no clear evidence where a block in the anthocyanin pathway might have occurred (Carvalho et al., 2013a), as no intermediate compounds or class of compounds were significantly accumulated in the turning stages (fruiting stage 3–4) of yellow and red fruiting raspberries.

### Regulation of Gene Expression

The anthocyanin accumulation in raspberry fruits becomes visible from ripening stage 3–4 (**Figure 1**). Therefore, expression analysis of the anthocyanin biosynthetic genes was carried out at these fruit developmental stages. As indicated in **Figure 1**, almost all structural anthocyanin genes show a significant increase in their expression; however, this was not the case for *Ans* in yellow fruits of “Anne”. The *Ans* transcripts were not induced in “Anne” when compared to “Tulameen” *Ans*. This reduction of *Ans* transcripts in “Anne” might be due to secondary effect of a nonsense mutation inside *Ans* gene. Such mechanism, known as nonsense-mediated mRNA decay (NMD), has been reported in plants (Schwartz et al., 2006; Wu et al., 2007) by which mutations inside a gene cause premature termination codons (PTCs) and quickly degrade mRNA to inhibit the accumulation of nonsense (inactive) proteins. Similar profiles with a reduced *Ans* expression among the anthocyanin pathway genes were also found in mock strawberry (Debes et al., 2011) and pomegranate (Zhao et al., 2015), leading to white fruiting anthocyanin-free phenotypes. In contrast, an unchanged expression of *Ans* was observed in the *Arabidopsis* loss-of function mutant *transparent testa 17* (*tt17*), where a SNP was found to result in an inactive protein and transparent testa seed phenotype (Appelhagen et al., 2011). However, our findings are in line with a recent study. In a white phenotypic fruit of pomegranates almost no expression of *Ans* gene was described. An insertion in coding sequence resulted in lack of *Pg-ldox* transcripts and of red pigments (Ben-Simhon et al., 2015). Similarly, in white fruited mock strawberry and yellow onion bulb no expression was observed of inactive *Ans* gene in comparison to active *Ans* gene in red phenotypes (Kim et al., 2004; Debes et al., 2011). Thus, reduced *Ans* transcripts might suggest a block at *Ans* level in “Anne”. As accumulation of flavonols and flavonol glycosides was observed in both, red and yellow raspberry fruits (Määttä-Riihinen et al., 2004; Carvalho et al., 2013a), similarly as they were found in the pale yellow Cactaceous and Caryophyllales species (Iwashina et al., 1988; Shimada et al., 2004) and later anthocyanin block was confirmed at *Ans* level (Shimada et al., 2005).

### Molecular Analysis of *Ans/ans* Alleles

The gene encoding ANS protein, a member of 2-ODD family has been reported for Rosaceae members and other plant species but not from *Rubus* yet. Among all 2-ODDs the catalytic residues are characterized by highly conserved histidine (His) residues for ferrous-iron coordination, and arginine (Arg) and serine (Ser) residues for binding site of 2-oxoglutarate and phenylalanine (Phe) residue for substrate binding (Saito et al., 1999; Koehntop et al., 2005; Clifton et al., 2006; Gebhardt et al., 2007; Cheng et al., 2014) as shown in **Figure 2**. Molecular analysis of *Ans* gene at genomic and mRNA/cDNA level indicates a 5 bp insertion (GGCCT; ans^+5^) in “Anne” *ans* alleles (**Figure 2**; Supplementary Figure S2). Furthermore, the coding region of “Anne” and “Tulameen” *Ans* contains a 446 bp intron (**Figure 2**). The *Rubus* intron comprises consensus “GT” and “AG” sequences at the 5′ and 3′ ends, respectively. This one intron genomic structure is similar to those found in *Fragaria* ×*ananassa, F. vesca, Malus, Allium, Theobroma, Arabidopsis, Forsythia, Prunus*, and *Petunia* (Weiss et al., 1993; Rosati et al., 1999; Deng and Davis, 2001; Almeida et al., 2007; Liu et al., 2013; Shen et al., 2014; Kim et al., 2015). The Ri-ANS protein sequence from “Tulameen” showed high (88%) identity to other plant species (*Malus* ANS, *Prunus* ANS, and *Pyrus* ANS) of Rosaceae family. The protein sequence is approximately 93% identical to *F. vesca* ANS, which is highly homologous to other functionally characterized plant ANSs. For example, it is 83, 78, and 73% identical to ANS from *Theobroma, Perilla*, and *Gerbera*, respectively. The known conserved domains were found in the deduced amino acid sequence of ANS from red fruiting raspberry “Tulameen”. However, the ans^+5^ in *Ans* gene of “Anne” led to a premature stop codon 20 aa upstream the insertion and the loss of the conserved His (iron binding site) and the RxS motif involved in 2-oxoglutarate binding. Furthermore, it has been described that as long the PTCs are present upstream of 3′ mRNA termini, they act as substrates (*cis*-acting elements) to activate NMD mechanism (Kertesz et al., 2006; Schwartz et al., 2006; Hori and Watanabe, 2007). Hence *Ans* PTC in “Anne” which is 455 bp distant from native 3′UTR, strongly supports this phenomenon.

### Molecular Marker for *Rubus* Screening

Molecular DNA- or RNA-based probe markers are common in use nowadays and have been reported for identification of SNPs or small target sequence within particular amplified fragments (Wittwer et al., 1997). Taking advantage of this technique, we designed a fluorogenic-labeled probe that proved to be useful for detection of mutants of *Ans* gene from genomic DNA and RNA/cDNA of “Anne” (**Figures 5A,B**). The probe developed here can further be utilized for characterization of specific insertion/mutation inside *Ans* gene to screen other yellow varieties of raspberry but can also be used as molecular marker for characterization of germplasm collections and offsprings in breeding programs.

### Complexity of *Ans* Gene

As the available genome draft of *Rubus* is not completed yet, it could not be excluded that more than one copy of *Ans* gene is present. Therefore, qPCR approach was performed to determine the complexity of the *Ans* gene, adopted of the method applied earlier to determine gene copy number in filamentous fungi (Solomon et al., 2008). The analysis indicated that there is only a single copy of the *Ans* gene in the genome of *R. idaeus* (as presented in **Table 1**), which is in agreement with the BLAST search of the *Rubus* genome draft (“Heritage”). The presence of a small gene family of the *Ans* gene has been reported for some species such as apple, peach, and diploid strawberry. Peach and octoploid strawberry (*Fragaria* ×*ananassa*) genome encodes two and four copies of *Ans* gene, respectively (Almeida et al., 2007; Ravaglia et al., 2013). Only one gene copy has been described in diploid strawberry (*F. vesca*) and apple (Takos et al., 2006; Lin-Wang et al., 2014).

### Functional Characterization of *Ans* Gene

In order to functionally characterize the *Rubus Ans* genes, transgenic lines were obtained through complementation of *ldox* mutant of *Arabidopsis* “ldox:KO” harboring *Ri-Ans* coding sequences from “Anne” and “Tulameen” expressed under the control of the constitutive *CaMV 35S* promoter. The plants transformed with “35S::Ans_Tulameen” showed a restored phenotype, while lines transformed with “35S::Ans_Anne” showed no changes when compared to “ldox:KO” line as shown in **Figure 4**. Chemical analysis confirmed the complementation of anthocyanin pathway due to presence of anthocyanins in transgenic plants transformed with “35S::Ans_Tulameen” (Supplementary Figure S3). However, no anthocyanins were detected from the plants transformed with “35S::Ans_Anne” like the “ldox:KO” line, similar to the well-established complementation reports (Abrahams et al., 2003; Teng et al., 2005; Solfanelli et al., 2006; Kovinich et al., 2014). These results confirm that “Tulameen-Ans” codes for a functional ANS protein while “Anne-Ans” results in a non-functional protein.

## Conclusion

**FIGURE 6:**
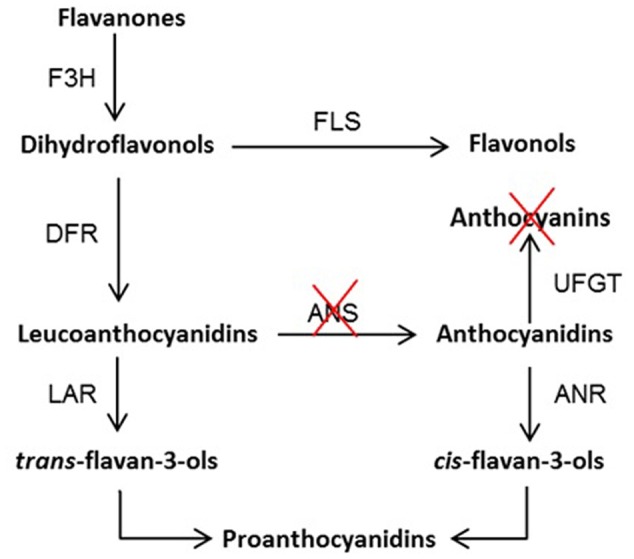
**Proposed flavonoid biosynthesis pathway in raspberry.** F3H, flavanone 3β-hydroxylase; DFR, dihydroflavonol 4-reductase; ANS, anthocyanidin synthase; UFGT, UDPG-flavonoid-glycosyltransferase; ANR, anthocyanidin reductase; LAR, leucoanthocyanidin reductase; and FLS, flavonol synthase. The red crosses indicate the blocked step of anthocyanin biosynthetic pathway at ANS level leading to the absence of anthocyanin pigments.

We identified a 5 bp insertion (ans^+5^) in *Ans* gene of yellow variety “Anne” as compared to red fruiting variety (“Tulameen”) of raspberry. We elucidated the functional role of ANS protein from “Tulameen” that imparts red fruiting color but is non-functional in yellow fruiting raspberry “Anne”. It is therefore considered that the ans^+5^ (GGCCT) in “Anne” generated a truncated polypeptide lacking the consensus motifs for cofactor and substrate binding, posing a complete loss-of-function in the mutant *Ri-Ans*. Deletion or insertion of a number of bases that is not a multiple of 3, usually introduces premature STOP codons in addition to lots of amino acid changes ultimately causing loss of function mutation of a protein sequence. We report a block in a step of anthocyanin pathway at ANS level in *Rubus* resulting in yellow anthocyanin-free fruit phenotype as indicated in the proposed pathway (**Figure 6**). It also supports the phenomenon that such nonsense mutations can abolish splicing resulting in NMD mechanism.

The probe developed in the current study can efficiently be utilized to screen other yellow and orange but also red varieties of raspberry for “Anne” like mutation inside *Ans* gene. In case of reduced anthocyanins, carotenoids are considered to be the main pigments accounting the pigmentation (yellow but also orange) in raspberries. Taken together, the data described here can further be utilized in breeding programs for indirect selection of genetic determinants of a trait of interest. Furthermore, other yellow and orange varieties are also under evaluation for possible flavonoid or carotenoid pathway blocks, eventually leading to develop a general pathway map for pigmentation in raspberry.

## Author Contributions

AF, EC, LH, and LP: Sample collection, preparation and expression analysis. EC and MR: *In silico* mining and cloning of *Ans* gene. LP and MR: *Ans* copy number analysis and development of probe-based marker. MM: Provided tissue culture facilities. MR and RS: Cloning, *Arabidopsis* transformation, and complementation. MR: Manuscript writing. SM: Overall guide as group leader and reviewed by all the co-authors.

## Conflict of Interest Statement

The authors declare that the research was conducted in the absence of any commercial or financial relationships that could be construed as a potential conflict of interest.
