# Supplementary material for: Nonsense Mutation Inside Anthocyanidin Synthase Gene Controls Pigmentation in Yellow Raspberry (Rubus idaeus L.)
Source: Front Plant Sci. 2016 Dec 19;7:1892. doi: 10.3389/fpls.2016.01892 (PMC5165238; doi:10.3389/fpls.2016.01892)
Supplement: Supplementary file 1 [file Table_1.DOCX]

Supplementary Table

**Nonsense mutation inside Anthocyanidin synthase gene controls pigmentation in yellow raspberry (*Rubus idaeus* L.).**

**Muhammad Zubair Rafique^1^, Elisabete Carvalho^1^, Ralf Stracke^2^, Luisa Palmieri^1^, Lorena Herrera^1^, Antje Feller^3^, Mickael Malnoy^1^ and Stefan Martens^1*^**

*** Correspondence:** Stefan Martens [Stefan.martens@fmach.it](mailto:Stefan.martens@fmach.it)

**Supplementary Table SI:** Primers designed for expression analysis and gene cloning purposes.

| Ans-utr-fwd | ATGCTCATTAAAGCATAACAAAGGCCC | Ans-utr-rev | TTAAACGGCTCCATTAATTAAGCAGCA |
| --- | --- | --- | --- |
| Ans-orf-fwd | CACCATGGTGACTGCTGCATCC | Ans-orf-rev | GCAGCATCTTATGTAGAGATGAGAGC |
| RubUni-fwd  FAM-probe | TGGAGAAGGAGGTCGGTGG  CTGCACTCACCTGGC | RubUni-rev | GGGAACCATGTTGTGGAGGAT |
| Chs-fwd | CCGACTACTACTTTCGTATCACCA | Chs-rev | ACTACCACCATGTCTTGTCTTGC |
| F3h-fwd | GTGCGCCACCGTGACTACTC | F3h-rev | ATGCCTTTGTCAATGCCTCC |
| Dfr-fwd | GGGTGGTGTTTACATCTTCGG | Dfr-rev | CTGCTTGCTCGGCTAGAGTTT |
| Ans-fwd | ATCGTCATGCACATAGGCGACACC | Ans-rev | CCTTGGGCGGCTCAGAGAAAA |
| Ufgt-fwd | ATCGTGGCTTGACAAACAGAA | Ufgt-rev | TGACCACAAGAATGGAACCCTA |
| Adh-fwd | TTGTGGAGAATACATGAACAAGG | Adh-rev | GAAACTGATCTAATGCTCCATGC |
