# Supplementary figures and images for: Nonsense Mutation Inside Anthocyanidin Synthase Gene Controls Pigmentation in Yellow Raspberry (Rubus idaeus L.)
Source: Front Plant Sci. 2016 Dec 19;7:1892. doi: 10.3389/fpls.2016.01892 (PMC5165238; doi:10.3389/fpls.2016.01892)

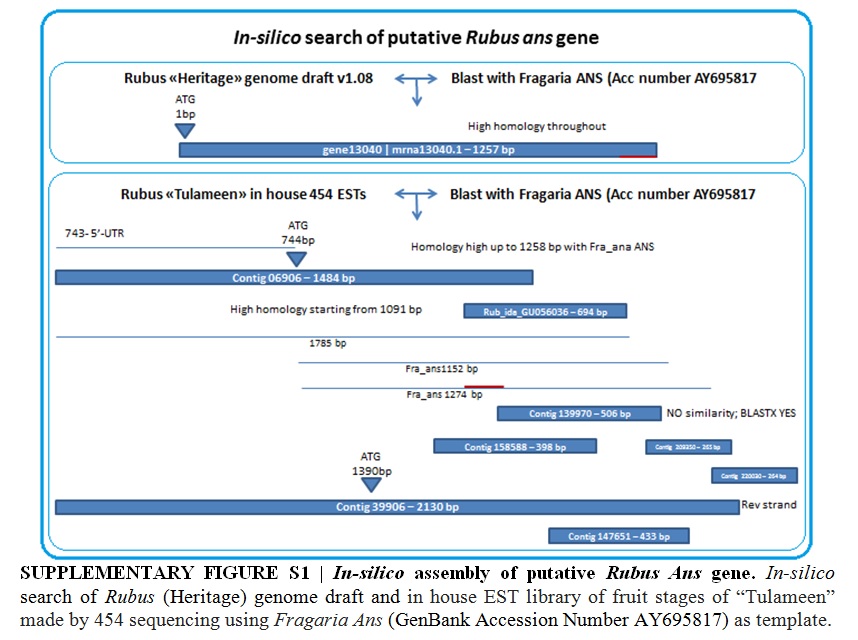

Supplement: Supplementary file 2 [file Image_1.JPEG]

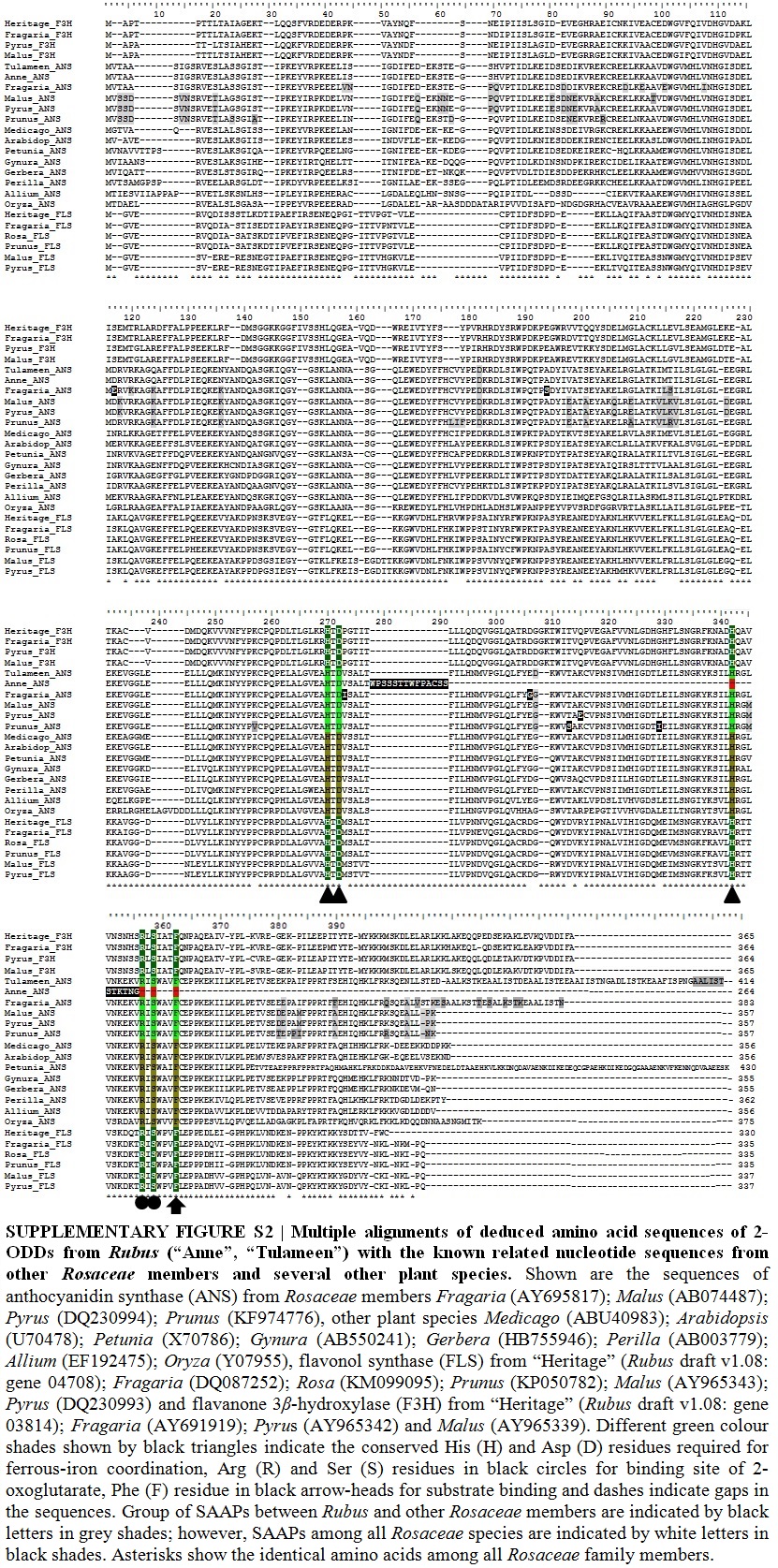

Supplement: Supplementary file 3 [file Image_2.JPEG]

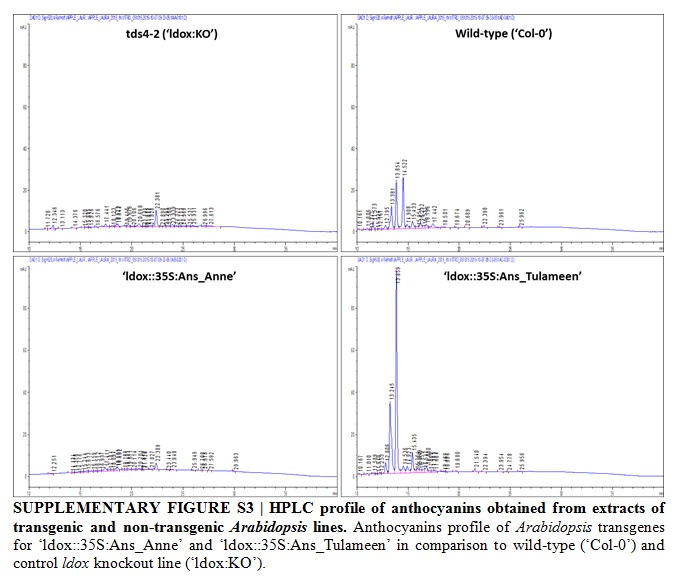

Supplement: Supplementary file 4 [file Image_3.JPG]
